# CD147 contributes to SARS-CoV-2-induced pulmonary fibrosis

**DOI:** 10.1038/s41392-022-01230-5

**Published:** 2022-11-25

**Authors:** Jiao Wu, Liang Chen, Chuan Qin, Fei Huo, Xue Liang, Xu Yang, Kui Zhang, Peng Lin, Jiangning Liu, Zhuan Feng, Jiansheng Zhou, Zhuo Pei, Yatao Wang, Xiu-Xuan Sun, Ke Wang, Jiejie Geng, Zhaohui Zheng, Xianghui Fu, Man Liu, Qingyi Wang, Zheng Zhang, Huijie Bian, Ping Zhu, Zhi-Nan Chen

**Affiliations:** 1 Department of Cell Biology of National Translational Science Center for Molecular Medicine and Department of Clinical Immunology of Xijing Hospital, Fourth Military Medical University, Xi’an, 710032 China; 2 Shanghai Engineering Research Center of Organ Repair, School of Medicine, Shanghai University, Shanghai, 200444 China; 3 Institute of Laboratory Animals Science, Chinese Academy of Medical Sciences, Beijing, 100071 China

**Keywords:** Infectious diseases, Molecular medicine

## Abstract

COVID‐19 patients can develop clinical and histopathological features associated with fibrosis, but the pathogenesis of fibrosis remains poorly understood. CD147 has been identified as a universal receptor for SARS-CoV-2 and its variants, which could initiate COVID-19-related cytokine storm. Here, we systemically analyzed lung pathogenesis in SARS-CoV-2- and its delta variant-infected humanized CD147 transgenic mice. Histopathology and Transmission Electron Microscopy revealed inflammation, fibroblast expansion and pronounced fibrotic remodeling in SARS-CoV-2-infected lungs. Consistently, RNA-sequencing identified a set of fibrosis signature genes. Furthermore, we identified CD147 as a crucial regulator for fibroblast activation induced by SARS-CoV-2. We found conditional knockout of CD147 in fibroblast suppressed activation of fibroblasts, decreasing susceptibility to bleomycin-induced pulmonary fibrosis. Meplazumab, a CD147 antibody, was able to inhibit the accumulation of activated fibroblasts and the production of ECM proteins, thus alleviating the progression of pulmonary fibrosis caused by SARS-CoV-2. In conclusion, we demonstrated that CD147 contributed to SARS-CoV-2-triggered progressive pulmonary fibrosis and identified CD147 as a potential therapeutic target for treating patients with post-COVID-19 pulmonary fibrosis.

## Introduction

Coronavirus disease 2019 (COVID-19), caused by infection of severe acute respiratory syndrome coronavirus-2 (SARS-CoV-2), continues to pose a severe global public health threat. Although most COVID-19 patients exhibited mild respiratory disease, the infection could lead to severe symptoms such as acute respiratory and pulmonary fibrosis.^1–3^

Pulmonary fibrosis is a chronic progressive disease characterized by excessive accumulation of extracellular matrix (ECM) within the lung interstitium and destruction of the parenchymal structure, leading to loss of pulmonary function.^4,5^ At the initiation stage of pulmonary fibrosis, stress and immune responses are triggered, followed by the activation of multiple proinflammatory pathways. Then, fibroblasts differentiate and proliferate at the proliferation stage. Finally, the ECM, which is composed of immune cells and fibroblast cells, is restructured in the modification stage. Multiple pieces of evidence indicated that long COVID-19 could lead to lung fibrosis. Computerized tomography (CT) scans of COVID-19 patients show ground-glass opacities in lungs that are prone to progression to patchy fibrosis.^3^ At the lung autopsy of fatal cases of COVID-19, pulmonary fibrotic changes have generally been found.^6^ In addition, a proteomic analysis of autopsy samples in COVID-19 patients revealed factors involved in fibrosis in multiple organs, including the lungs.^7^ It has also been suggested that COVID-19 patients are at increased risk of developing fibrotic disease and long-term damage even after virus eradication.^6^ For the development of effective symptomatic treatments, the potential mechanism underlying the fibroblast-to-myofibroblast transition associated with COVID-19 pulmonary fibrosis needs further exploration.

CD147, an adhesion molecule, has been shown to be an important mediator of inflammatory and immune responses. Our previous studies have identified the CD147-spike protein as a novel and universal route for the infection of SARS-CoV-2 and its variants in host cells.^8,9^ As a signaling transducer, CD147 also contributes to cytokine storm syndromes (CSSs) in severe COVID-19 cases by its ability to regulate the expression of CyPA.^9^ CSSs are usually characterized by elevated serum levels of proinflammatory cytokines and chemokines and play an important role in the process of disease aggravation, causing pulmonary and multiorgan injury, especially acute inflammatory fibrotic lung disease.^10,11^ However, it remains unclear whether CD147-mediated CSSs is associated with the pulmonary fibrosis. Here, we identified a novel function of CD147 contributing to fibroblasts activation in COVID-19 pulmonary fibrosis besides mediating entry of virus and inducing cytokine storm. Humanized CD147 (hCD147) transgenic mouse model with SARS-CoV-2 infection was found to mimic pulmonary fibrosis progression. CD147 was identified as a crucial regulator for fibroblasts activation induced by SARS-CoV-2 and bleomycin. The humanized anti-CD147 antibody, meplazumab, could inhibit the accumulation of fibroblasts and production of ECM proteins, thus alleviating the progression of pulmonary fibrosis caused by SARS-CoV-2. Overall, these findings improve our understanding of the pulmonary fibrosis secondary to COVID-19 and provide avenues for developing strategies for the treatment of fibrotic symptoms.

## Results

### A humanized CD147 transgenic mouse model with SARS-CoV-2 and delta variant infection mimics pulmonary fibrosis pathology

**Fig. 1:**
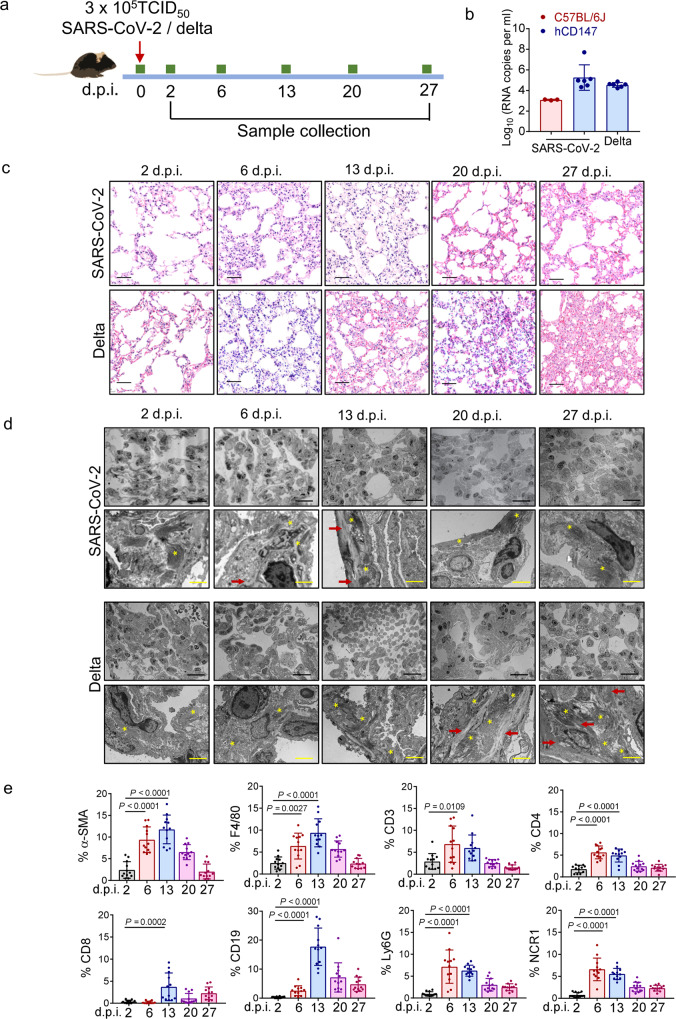
hCD147 transgenic mice infected with SARS-CoV-2 and delta variant mimicked pulmonary fibrosis pathology. **a** hCD147 mice or C57BL/6J mice were inoculated via the intranasal with 3 × 10^5^ TCID_50_ of SARS-CoV-2 or delta variant, and samples were collected at 2, 6, 13, 20 and 27 d.p.i. **b** RT-qPCR for viral RNA levels in lung tissues at 2 d.p.i. *n* = 3 for C57BL/6J group, *n* = 6 for hCD147 groups. **c** H&E staining of lung tissue sections from 2 to 27 d.p.i. Scale bars, 50 μm. **d** TEM analysis of lung samples from 2 to 27 d.p.i. The stars indicate collagen fibrils; the arrows indicate elastic fibers. Black scale bars, 20 μm. Yellow scale bars, 2 μm. **e** Statistics of the percentage of cells positive for α-SMA (fibroblasts), F4/80 (macrophages), CD3 (T cells), CD4, CD8, CD19 (B cells), Ly6G (neutrophils), and NCR1 (NK cells). *n* = 12 images for each group, one-way ANOVA followed by multiple comparisons

In our previous study, a humanized CD147 (hCD147) transgenic mouse model was constructed to investigate the mechanisms by which COVID-19 affects the endocrine and immune systems.^9^ Lung tissues of SARS-CoV-2-infected mice showed accumulation of inflammatory cells and elevated levels of cytokines and chemokines at 6 days post-infection (d.p.i.), suggesting that SARS-CoV-2 could induce endocrine dyscrasia and strong immune responses through CD147.^9^ To investigate the long-term damage and the occurrence of pulmonary fibrosis caused by SARS-CoV-2, we inoculated hCD147 mice via the intranasal route with 3 × 10^5^ TCID_50_ of SARS-CoV-2 or the delta variant and extended the time points to 27 d.p.i. (Fig. 1a). High levels of viral RNA were detected in lung tissues at 2 d.p.i., confirming the successful infection of hCD147 mice with SARS-CoV-2 or its delta variant (Fig. 1b). However, the level of viral RNA was much lower in wild type mice (C57BL/6J) (Fig. 1b). Since the CD147-spike protein has been identified as a novel route for the infection of SARS-CoV-2, we performed docking and molecular dynamics simulation to further understand the details of the interaction between human/mouse CD147 and the spike protein (Supplementary Fig. 1a). Analysis showed 13 pairs of hydrogen bonds formed between human CD147 and receptor binding domain (RBD) of the spike protein, in which 10 out of 13 hydrogen bonds may be disrupted in mouse CD147 because of residue substitutions (Supplementary Fig. 1b). The sequence alignment of human and mouse CD147 also showed a relatively low homology (Supplementary Fig. 1c, amino acid identity = 57.9%). To measure the binding affinities between human/mouse CD147 and RBD of the spike protein, surface plasmon resonance (SPR) was performed and showed an interaction between human CD147 and RBD, with an affinity constant of 4.47 × 10^−6^ M (Supplementary Fig. 1d). However, no binding signals were detected between mouse CD147 and RBD (Supplementary Fig. 1d), which was consistent with the molecular docking results.

H&E staining of lung sections showed distinctive pathological characteristics at different time points. At 6 d.p.i. and 13 d.p.i., lung tissues of hCD147 mice with SARS-CoV-2 developed pathological changes characterized by alveolar septal thickening, infiltration of macrophages, neutrophils and lymphocytes, and pulmonary consolidation (Fig. 1c). However, similar pathological changes in lung tissues of mice with the delta variant tended to be more persistent than those of mice infected with SARS-CoV-2 (Fig. 1c). Transmission electron microscopy (TEM) analysis of lung samples revealed thickened alveolar septa and an accumulation of collagen fibrils and elastic fibers (Fig. 1d).

At 6 days and 13 days after SARS-CoV-2 infection, immunofluorescence analyses showed that the population of α-SMA^+^ fibroblasts was increased (Fig. 1e, Supplementary Fig. 2). Since both innate and adaptive immune cell responses have been linked to fibroblast activation and fibrogenesis in pulmonary fibrosis, we analyzed the population of main immune cell types. A significant increase in the percentages of macrophages, T cells, B cells, neutrophils, and NK cells was observed in lung tissue, which peaked at 6 d.p.i. or 13 d.p.i. (Fig. 1e, Supplementary Fig. 2). These pathological changes in SARS-CoV-2-infected lungs were consistent with pulmonary fibrosis, which is characterized by interstitial fibroblast proliferation and deposition of ECM proteins, particularly collagen. Notably, the expansion of fibroblasts and infiltration of inflammatory cells were weaker in the lungs at 20 d.p.i. and 27 d.p.i. than that at 14 d.p.i., indicating the gradual abolishment of inflammation and fibrotic remodeling. Consistently, in the majority of COVID-19 survivors, fibrotic tissue remodeling occurs rapidly and is at least partially reversible.^12^ Since SARS-CoV-2-induced injuries and dysregulation of angiogenesis, coagulation and fibrosis have been detected in multiple organs,^13,14^ we collected and stained other main organs, including the heart, liver, spleen and kidney. While no obvious pathological changes in these organs of SARS-CoV-2-infected mice were observed, TEM analysis of kidney samples revealed an accumulation of collagen fibrils (Supplementary Fig. 3), consistent with the growing recognition of renal dysfunction and fibrosis in the COVID-19 patients.^7,15^

### SARS-CoV-2 causes stronger fibrotic remodeling phenotypes in the hCD147 mouse model than in the hACE2 mouse model

ACE2 is one of the important receptors mediating SARS-CoV-2 infection of host cells by recognition of the spike protein.^16^ Our previous study indicated that SARS-CoV-2 causes much stronger immune responses through CD147 than ACE2, which triggers the cytokine storm.^9^ Here, we compared the pathology and fibrotic features between the hACE2 and hCD147 mouse models infected with SARS-CoV-2. Lung tissues of SARS-CoV-2-infected hACE2 mice developed pathological changes characterized by alveolar septal thickening (Supplementary Fig. 4a). However, accumulation of collagen fibrils and elastic fibers was much milder than hCD147 mice under the electron microscope (Supplementary Fig. 4b). Although the infiltration of macrophages and T cells were similar in both mice at 6 d.p.i. and 13 d.p.i., the expansion of α-SMA^+^ fibroblasts was rarely observed in alveoli of hACE2 mice (Supplementary Fig. 4c).

### The hCD147 mouse model with SARS-CoV-2 infection showed fibrotic transcriptional signatures similar to those associated with classical pulmonary fibrosis revealed by RNA-sequencing (RNA-seq) analysis

**Fig. 2:**
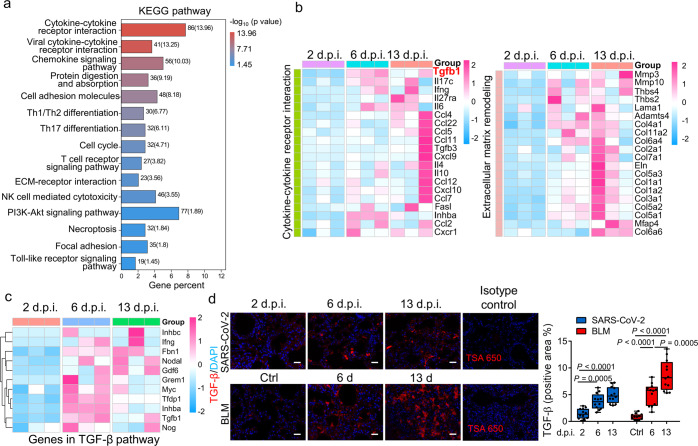
hCD147 mice with SARS-CoV-2 infection showed fibrotic characteristics revealed by RNA-seq analysis. **a** KEGG enrichment analysis of pathways enriched in differentially expressed genes in three profiles showing increasing trends. **b** Heatmap of significantly enriched genes involved in cytokine-cytokine receptor interaction and ECM remodeling in the three profiles. **c** Heatmap of significantly enriched genes involved in TGF-β pathway. **d** Immunofluorescence staining and quantification of TGF-β in lung tissue sections from SARS-CoV-2-infected hCD147 mice and bleomycin-induced pulmonary fibrosis model at indicated time points. The rabbit IgG isotype control was used along with the staining of TGF-β. Scale bars, 50 μm. *n* = 12 images for each group, one-way ANOVA followed by multiple comparisons

To further investigate the pathogenesis of pulmonary fibrosis in a SARS-CoV-2-infected hCD147 mouse model, RNA-seq was performed on the lung tissues at 2, 6, and 13 d.p.i. We analyzed the gene enrichment trends and found three different profiles: profile #1 (continuous increase from 2 to 13 d.p.i.), profile #2 (starting to increase from 6 d.p.i.) and profile #3 (reaching a plateau at 6 d.p.i.) (Supplementary Fig. 5). Kyoto Encyclopedia of Genes and Genomes (KEGG) enrichment showed that multiple immune-related signals displayed increasing trends, including cytokine signaling, chemokine signaling, Th1- and Th2-cell differentiation, Th17-cell differentiation signaling, T cell receptor signaling and NK-cell-mediated cytotoxicity (Fig. 2a). In the pathogenesis of pulmonary fibrosis, immune cells modulate fibrogenesis through various mechanisms.^17^ Emerging evidences reveal that the Th1/Th2 balance plays a modulatory role during the inflammatory phase of pulmonary fibrosis.^18^ Th2 secreted cytokines such as IL-4, IL-5 and IL-13 that promote pulmonary fibrosis, whereas Th1 cytokines IFN-γ and IL-12 inhibit fibrogenesis.^19,20^ Previous studies also support a role for Th17 cells and its cytokine IL-17 in different murine pulmonary fibrosis models, since neutralization of IL-17A promoted the resolution of fibrosis.^21,22^

Moreover, genes enriched in pathways representing ECM remodeling and fibrosis, including ECM-receptor interaction signaling, protein digestion and absorption signaling were increased (Fig. 2b). Several secretory proteins involved in deposition of ECM proteins and tissue remodeling were upregulated, including proteases (MMP3, MMP10, ADAMTS4) and ECM proteins (COL1A1, COL1A2, COL2A1, COL3A1, COL5A1, COL5A2, ELN, MFAP4, THBS4, etc.) (Fig. 2c). Apart from many elevated cytokines and chemokines exerting profibrotic functions in fibrogenesis (IL-4, IL-6, IL-17, IFN-γ, CCL2, CCL4, IL-10, CXCL9, CXCL10, etc.), the main profibrogenic cytokine TGF-β and activation of TGF-β pathway were found to increase in lungs of SARS-CoV-2-infected hCD147 mouse model (Fig. 2b, c). During the initiation of the pulmonary fibrotic phase, epithelial cell injury and the activation of inflammation promote the production of TGF-β, which plays a critical role in the process by which fibroblasts differentiate into myofibroblasts, produce collagen, reduce lung elasticity and impair respiratory function.^23,24^ We induced a pulmonary fibrosis model in mice with bleomycin, which is commonly applied for pulmonary fibrosis modeling in rodents.^25–27^ Immunofluorescence verified the elevation of TGF-β in the lung tissues of SARS-CoV-2-infected hCD147 mice and the bleomycin-induced pulmonary fibrosis model mice (Fig. 2d).

To compare the gene signatures between SARS-CoV-2-infected hCD147 mice and the classical pulmonary fibrosis model, RNA-seq was performed on normal lung tissues and at 6 days and 13 days post-bleomycin administration. We found a high similarity of gene expression in lung tissues of hCD147 mice infected with SARS-CoV-2 and C57BL/6J mice treated with bleomycin. Trend analysis and KEGG enrichment showed that immune-related signals and potent fibrosis-related pathways displayed similar increasing trends (Supplementary Fig. 6a–d). In addition, the transcriptional profile of fibrosis-associated key cytokines and secretory proteins involved in the deposition of ECM proteins and tissue remodeling showed similarity to SARS-CoV-2-infected lungs (Supplementary Fig. 6e). All these results indicate that SARS-CoV-2 could trigger enrichment of the fibrotic transcriptional profile in the hCD147 mouse model, highlighting hCD147 mice as an ideal model for studying the fibrotic pathogenesis of COVID-19.

### The TGF-β-CD147 axis contributes to fibroblast activation in the lungs of a SARS-CoV-2-infected hCD147 mouse model

**Fig. 3:**
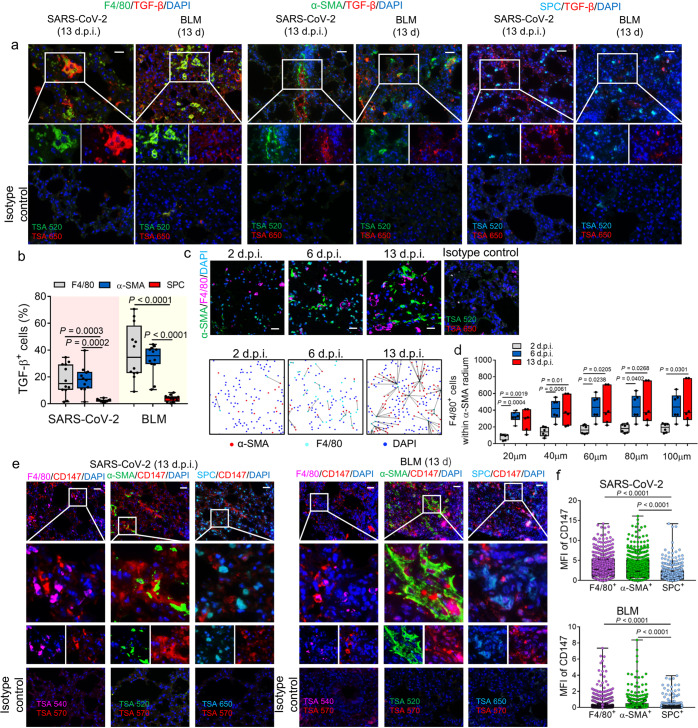
Activation of TGF-β-CD147 axis in fibroblasts in lungs of SARS-CoV-2-infected hCD147 mouse model. **a** Multiplex immunohistochemistry of lung tissue sections for F4/80, α-SMA, SPC and TGF-β from SARS-CoV-2-infected hCD147 mice at 13 d.p.i. and bleomycin-induced pulmonary fibrosis model at 13 days post-bleomycin administration. Scale bars, 50 μm. **b** Statistics of the percentages of cells expressing TGF-β in cells positive for F4/80, α-SMA or SFTPC (SPC). *n* = 12 images from three mice for each group, one-way ANOVA followed by multiple comparisons. **c** Representative multiplex immunohistochemistry staining of lung tissue sections from SARS-CoV-2-infected hCD147 mice for F4/80 and α-SMA from 2 to 13 d.p.i. Scale bars, 25 μm. **d** The number of F4/80^+^ macrophages within the 100 μm radium of α-SMA^+^ fibroblasts. *n* = 6 images (100 ×) from 3 mice for each group, one-way ANOVA followed by multiple comparisons. **e** Multiplex immunohistochemistry of lung tissue sections for CD147, F4/80, α-SMA and SPC from SARS-CoV-2-infected hCD147 mice at 13 d.p.i. and bleomycin-induced pulmonary fibrosis model at 13 days post-bleomycin administration. For samples from SARS-Cov2-infected hCD147 mice and bleomycin-treated C57BL/6J mice, anti-human CD147 antibody and anti-mouse CD147 antibody were used respectively. Scale bars, 50 μm. **f** Quantification of the mean fluorescence intensity (MFI) of CD147 in cells positive for F4/80, α-SMA or SPC. One-way ANOVA followed by multiple comparisons. In **a**, **c** and **e**, the rabbit or mouse IgG isotype control was used along with the staining of each molecule

One reasonable explanation for SARS-CoV-2-induced pulmonary fibrosis-like characteristics in the hCD147 mouse model is that CD147 serves as a potent receptor for the virus and a regulator of strong immune responses, leading to production of profibrotic cytokines and causing expansion of fibroblasts and myofibroblasts. However, as a transmembrane glycoprotein, CD147 was also reported to stimulate hepatic stellate cell (HSC) activation and proliferation by a TGF-β1-CD147 self-sustaining network, promoting the upregulation of fibrogenesis-related genes and liver fibrosis.^28,29^ We then focused on the possible contribution of CD147 to the activation of fibroblasts in pulmonary fibrosis in addition to the already established functions in COVID-19 pathogenesis. Since the master regulator of organ fibrosis, TGF-β, was shown to be highly expressed in both SARS-CoV-2-infected lung tissues and classical pulmonary fibrotic lung tissues, we analyzed the TGF-β expression on macrophages, fibroblasts and type II alveolar epithelial cells (AEC2), which are widely accepted as key effector cells that generate TGF-β in idiopathic pulmonary fibrosis.^30^ TGF-β was found mainly expressed on infiltrated macrophages and fibroblasts in both SARS-CoV-2-infected hCD147 mice at 13 d.p.i. and pulmonary fibrosis model at 13 days post-bleomycin administration (Fig. 3a, b). Interestingly, the number of F4/80^+^ macrophages within the 100 μm radius of α-SMA^+^ fibroblasts gradually increased from 2 d.p.i. to 13 d.p.i. in the lungs of SARS-CoV-2-infected hCD147 mice (Fig. 3c, d). Given the evidence that pulmonary macrophages in severe COVID-19 express a range of genes associated with profibrotic functions including TGF-β and genes related to TGF-β signaling,^12^ it is reasonable to assume that macrophage-fibroblast intercellular communication might promote the pronounced expansion and activation of myofibroblasts and fibroblasts through TGF-β signaling. We then detected the expression of CD147 in the lung tissues, and found that CD147 showed higher expression levels on α-SMA^+^ fibroblasts and F4/80^+^ macrophages compared with AEC2 cells at 13 d.p.i. (Fig. 3e, f). The expression of CD147 in different cell types was consistent with that of bleomycin-induced mice at 13 days (Fig. 3e, f). These results indicate a potential role of CD147 expressed on fibroblasts in fibrogenesis induced by SARS-CoV-2 infection.

**Fig. 4:**
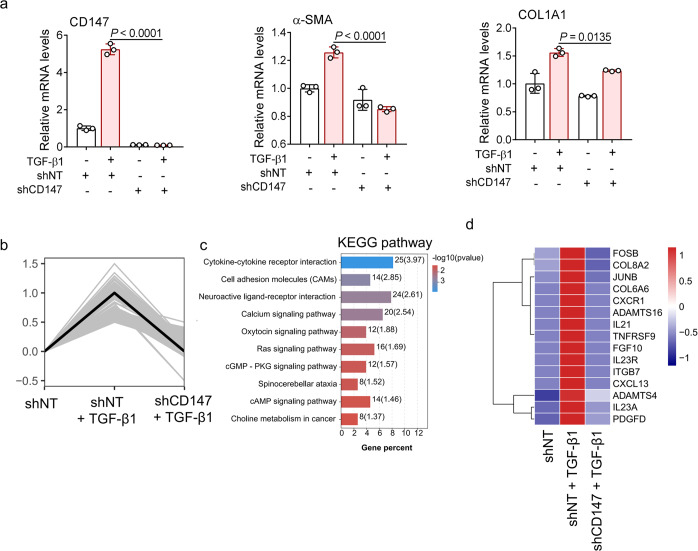
CD147 knockdown inhibits activation of lung fibroblasts upon TGF-β stimulation. **a** MRC-5 cells were transfected with shRNA for CD147 gene silence and stimulated with TGF-β (10 ng/mL) for 24 h. The gene expression levels of CD147, α-SMA and COL1A1 were detected by real-time PCR. *n* = 3 samples for each group, one-way ANOVA followed by multiple comparisons. **b** The statistically significant profile by trend analysis of differentially expressed genes in MRC-5 cells transfected with CD147 shRNA with or without stimulation of TGF-β. **c** KEGG enrichment analysis of pathways enriched in the profile in **b**. **d** Heatmap of significantly enriched genes in the profile. Columns represent samples and rows represent genes. Gene expression levels in the heat maps are z score–normalized values determined by log_2_^[CPM]^ values

To clarify whether the activation of fibroblasts involves the TGF-β-CD147 axis, we stimulated human lung fibroblast cell line MRC-5 with TGF-β and measured the expression of CD147 and fibroblast activation markers, including α-SMA and COL1A1. TGF-β upregulated the expression levels of CD147, α-SMA and COL1A1, which were markedly suppressed by CD147 knockdown (Fig. 4a). RNA-seq was performed to analyze enriched genes that were increased upon TGF-β stimulation but decreased with CD147 knockdown (Fig. 4b). KEGG enrichment showed that the increasing trend of cytokine-cytokine receptor interaction signaling induced by TGF-β was attenuated by depletion of CD147 (Fig. 4c). In addition, several secretory proteins involved in fibrogenesis and tissue remodeling upregulated by TGF-β were decreased by the loss of CD147, including fibrosis-associated cytokines (IL-21, IL-23, FGF, PDGF-D), proteases (ADAMTS4, ADAMTS16), and ECM proteins (COL6A6, COL8A2) (Fig. 4d). Collectively, these data confirmed the contribution of the TGF-β-CD147 axis to fibroblast activation. Previous studies revealed that activated CD44 can promote the process of TGF-β-induced myofibroblast differentiation by interacting with CD147.^31^ TGF-β stimulation greatly increased the co-localization of CD147 and CD44 on the plasma membrane in MRC-5 cells (Supplementary Fig. 7a). However, knockdown of CD44 failed to suppress the expression levels of CD147 and fibroblast activation markers including α-SMA, COL1A1, COL1A2, MMP2 and MMP9 induced by TGF-β (Supplementary Fig. 7b, c). These results indicate that TGF-β-CD147 axis could contribute to fibroblast activation in a CD44-independent manner.

### Conditional deletion of CD147 in fibroblasts reverses pulmonary fibrosis in the bleomycin model

**Fig. 5:**
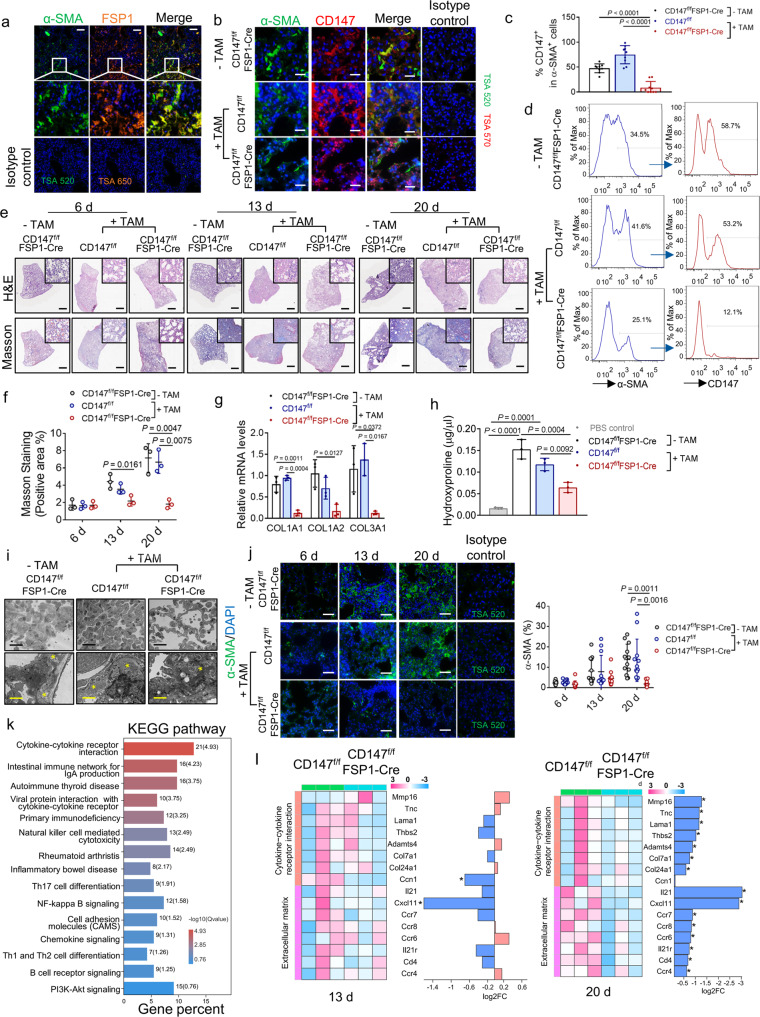
Ablation of CD147 in fibroblasts reverses pulmonary fibrosis in the bleomycin model. **a** Representative multiplex immunohistochemistry staining of lung tissue sections from bleomycin-induced pulmonary fibrosis model (13 d) for α-SMA and FSP1. Scale bars, 200 μm. **b** Representative multiplex immunohistochemistry staining of lung tissue sections from bleomycin-induced CD147^f/f^FSP1-Cre and CD147^f/f^ mice treated with or without tamoxifen (TAM) for α-SMA and CD147. Scale bars, 50 μm. **c** Statistics of the percentages of cells expressing CD147 in cells positive for α-SMA. *n* = 9 images from three mice for each group, one-way ANOVA followed by multiple comparisons. **d** Percentages of α-SMA fibroblasts^+^ and CD147-expressing α-SMA^+^ fibroblasts detected by flow cytometry in lung tissues of bleomycin-induced pulmonary fibrosis model. **e** H&E staining and Masson’s trichrome staining of lung tissues from CD147^f/f^FSP1-Cre and CD147^f/f^ mice treated with or without tamoxifen at indicated time points after bleomycin administration. Scale bars, 1 cm. **f** Statistics of the positive area of Masson’s trichrome staining. *n* = 3 mice for each group, one-way ANOVA followed by multiple comparisons. **g** Gene expression of COL1A1, COL1A2 and COL3A1 in lung homogenates determined by RT-qPCR. GAPDH is used as a reference gene. **h** Hydroxyproline content from CD147^f/f^FSP1-Cre and CD147^f/f^ mice treated with or without tamoxifen at 13 days after bleomycin administration. CD147^f/f^ mice receiving PBS serves as control group. *n* = 3 mice for each group, one-way ANOVA followed by multiple comparisons. **i** TEM analysis of lung tissues from bleomycin-induced CD147^f/f^FSP1-Cre and CD147^f/f^ mice at indicated time points. Black scale bars, 20 μm; yellow scale bars, 2 μm. The stars indicate collagen fibrils. **j** Immunofluorescence staining and quantification of α-SMA in lung tissue sections from bleomycin-induced CD147^f/f^FSP1-Cre and CD147^f/f^ mice at indicated time points. Scale bars, 100 μm. *n* = 12 images from three mice for each group, one-way ANOVA followed by multiple comparisons. **k** KEGG enrichment analysis of pathways enriched in downregulated genes in lungs of CD147^f/f^FSP1-Cre mice compared with CD147^f/f^ mice. **l** Heatmap of genes associated with cytokines-cytokine receptor interaction and ECM remodeling at indicated time points. In panel **a**, **b** and **j**, the rabbit or mouse IgG isotype control was used along with the staining of each molecule

Fibroblast-specific protein 1 (FSP1, also referred to as S100A4) is considered as a marker of fibroblasts in different organs undergoing tissue remodeling and could serve as a sensitive and specific marker for lung fibroblasts.^32^ As shown in Fig. 5a, multiplex immunohistochemistry staining showed a large but not complete overlapping of FSP1 and α-SMA in fibrotic lung tissue. To determine the role of CD147 in the activation of fibroblasts in pulmonary fibrosis progression, we generated mice that lack CD147 selectively in fibroblasts. We crossed mice expressing a conditional CD147 knockout allele conditional (CD147^f/f^) with mice that express Cre recombinase driven by the FSP1 promoter (FSP1-CreERT2). The offspring are referred to hereafter as CD147^f/f^FSP1-Cre, in which CD147 in FSP1^+^ cells could be conditionally knocked out after consecutive intraperitoneal injection of tamoxifen. Tamoxifen was intraperitoneally injected for 5 days to induce CD147 knockout. From the 6th day after tamoxifen administration, the bleomycin-induced pulmonary fibrosis model was established in CD147^f/f^FSP1-Cre mice and control mice (CD147^f/f^FSP1-Cre mice without tamoxifen treatment and CD147^f/f^ with tamoxifen treatment). CD147^f/f^ mice receiving the same volume of phosphate buffered saline (PBS) showed no sign of alveolar structure destroying, lung interstitium thickening or increased deposition of collagen, measured by H&E staining and Masson’s trichrome staining (Supplementary Fig. 8). α-SMA and CD147 in the lung tissues of all groups were labeled by multiplex immunohistochemistry at 13 days after bleomycin administration. As shown in Fig. 5b, c, the staining of CD147 in α-SMA^+^ fibroblasts of tamoxifen-treated CD147^f/f^FSP1-Cre mice was markedly decreased compared to that of untreated CD147^f/f^FSP1-Cre mice and tamoxifen-treated CD147^f/f^ mice. Flow cytometry analysis showed that tamoxifen treatment decreased the population of α-SMA^+^ fibroblasts and the frequency of CD147^+^ cells in α-SMA^+^ fibroblasts in CD147^f/f^FSP1-Cre mice, confirming the deletion of CD147 in lung fibroblasts (Fig. 5d). Comparing lung tissue at 6, 13, and 20 days after bleomycin administration, we found that pathological changes of fibrosis of CD147^f/f^FSP1-Cre mice were alleviated at day 13 and day 20 compared to tamoxifen-treated CD147^f/f^ mice and CD147^f/f^FSP1-Cre mice without tamoxifen treatment (Fig. 5e). H&E staining demonstrated that conditional knockout of CD147 in fibroblasts alleviated the pathological changes of bleomycin-induced pulmonary fibrosis, which was particularly significant in the later stage of fibrosis (Fig. 5e). Masson’s trichrome staining showed that collagen deposition was relatively reduced in lung tissues of CD147-knockout mice (Fig. 5e, f). Moreover, conditional knockout of CD147 markedly inhibited the levels of ECM remodeling proteins such as COL1A1, COL1A2 and COL3A1 at 13 days after bleomycin administration (Fig. 5g). We also detected the hydroxyproline content to assess collagen deposition in lung tissue of each group. Knockout of CD147 in fibroblasts strongly suppressed the level of hydroxyproline content in lung tissues (Fig. 5h). TEM analysis of lung samples revealed that thickened alveolar septa and accumulation of collagen fibrils were alleviated in lung tissues of CD147conditional knockout mice compared with control mice (Fig. 5i). The expression of α-SMA was also decreased in bleomycin-induced fibrotic lung tissues of CD147-conditional knockout mice (Fig. 5j).

To further confirm the attenuated occurrence of pulmonary fibrosis by fibroblast-specific knockout of CD147, we performed RNA-seq using lung tissues at 13 days and 20 days post-bleomycin induction. KEGG analysis of the downregulated genes in the lungs of CD147^f/f^FSP1-Cre mice compared with CD147^f/f^ mice, and the results showed that these genes were enriched in pathways related to the pathogenesis of pulmonary fibrosis, including cytokine/chemokine-related signaling, Th17-cell differentiation signaling, Th1-/Th2-cell differentiation and NK-cell-mediated cytotoxicity (Fig. 5k). It is widely accepted that the bleomycin-induced lung injury model is composed of two phases: an early inflammatory phase characterized by infiltration of inflammatory cells into the lungs, followed by a late fibrotic phase characterized by increased fibroblast proliferation and differentiation to myofibroblasts, as well as synthesis of ECM.^25,33,34^ The downregulated profibrotic cytokines and ECM proteins were mainly enriched in the lungs at 20 days post-establishment of the fibrosis model, indicating the crucial role of CD147 on fibroblast activation in the late fibrotic phase (Fig. 5l). Taken together, these results demonstrate that CD147 expression in fibroblasts effectively promotes the activation of fibroblasts and the occurrence of pulmonary fibrosis. Conditional deletion of CD147 in fibroblasts could prevent the fibrotic progression.

### CD147 antibody alleviates pulmonary fibrosis caused by SARS-CoV-2

**Fig. 6:**
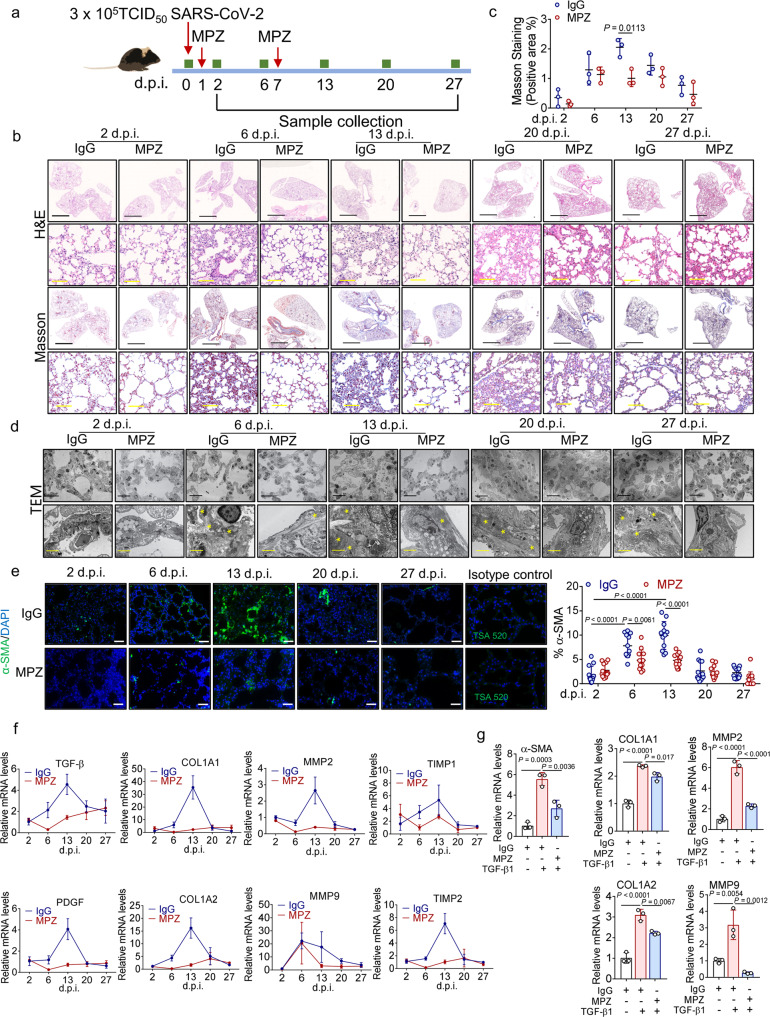
Meplazumab prevents pulmonary fibrosis caused by SARS-CoV-2. **a** hCD147 mice were inoculated via the intranasal with 3 × 10^5^ TCID_50_ of SARS-CoV-2 and treated with CD147 antibody meplazumab at 1 d.p.i. and 7 d.p.i. Samples were collected at 2, 6, 13, 20 and 27 d.p.i. **b** H&E staining and Masson’s trichrome staining of lung tissue sections from the IgG and meplazumab groups at different time points. Black scale bars, 2 mm. Yellow scale bars, 100 μm. **c** Statistics of the positive area of Masson’s trichrome staining. *n* = 3 mice for each group, two-tailed unpaired *t*-test. **d** TEM analysis of lung tissues from the IgG and meplazumab groups. Black scale bars, 20 μm; yellow scale bars, 2 μm. The stars indicate collagen fibrils. **e** Immunofluorescence staining and quantification of α-SMA in lung tissue sections from the IgG and meplazumab groups. Scale bars, 100 μm. *n* = 12 images from three mice for each group, one-way ANOVA followed by multiple comparisons. **f** Gene expression of TGF-β, PDGF, MMP2, MMP9, TIMP1, TIMP2, COL1A1 and COL1A2 in lung homogenates determined by RT-qPCR, compared with the corresponding IgG controls at indicated time points. GAPDH is used as a reference gene. **g** Gene expression of α-SMA, COL1A1, COL1A2, MMP2 and MMP9 in MRC-5 cells pre-incubated with IgG or meplazumab (60 μg/mL) followed by stimulation of TGF-β (10 ng/mL), determined by RT-qPCR. GAPDH is used as a reference gene

To verify whether CD147 also functions as a therapeutic target for COVID-19-induced pulmonary fibrosis, we treated SARS-CoV-2 infected hCD147 mice with meplazumab (Fig. 6a). The results showed that meplazumab effectively attenuated the pathological changes of pneumonia at 6 d.p.i. Strikingly, the pathological changes of pulmonary fibrosis characterized by alveolar septal thickening and pulmonary consolidation were relieved (Fig. 6b). Masson’s trichrome staining showed that meplazumab significantly reduced bleomycin-induced collagen production in the lung at 13 d.p.i. (Fig. 6b, c). TEM analysis of lung samples revealed decreased accumulation of collagen fibrils after meplazumab treatment at 6 d.p.i. and 13 d.p.i. (Fig. 6d). At 6 days and 13 d.p.i. with SARS-CoV-2, immunofluorescence analyses showed that meplazumab reduced the population of α-SMA^+^ fibroblasts (Fig. 6e). Moreover, meplazumab treatment markedly inhibited the levels of profibrotic cytokines, such as TGF-β and PDGF, and the production of ECM remodeling proteins such as COL1A1, COL1A2, MMP2, MMP9, TIMP1 and TIMP2 at 6 d.p.i. and 13 d.p.i. (Fig. 6f). We then pre-incubated human lung fibroblast cell line MRC-5 with meplazumab and stimulated the cells with TGF-β. TGF-β upregulated the expression levels of α-SMA, COL1A1, COL1A2, MMP2 and MMP9 in fibroblasts, which were markedly suppressed by meplazumab (Fig. 6g). Taken together, these results showed that meplazumab can effectively alleviate SARS-CoV-2-induced pulmonary fibrosis, demonstrating that CD147 is a promising early intervention target for antifibrotic therapies.

## Discussion

The lungs are the primary source of infection and the most vulnerable tissue in COVID-19.^35,36^ In the lungs of individuals who have died of COVID-19, features of bilateral diffuse alveolar damage, signs of respiratory inflammation and fibrosis have been identified.^35,37^ Although various mechanisms of lung injury in COVID-19 have been described, pathomechanisms of COVID-19-associated lung fibrosis remain incompletely understood. Previous studies mainly focus on COVID-19 patients, whereas an ideal animal model for studying the pathogenesis and potential therapeutic strategies of COVID-19-associated lung fibrosis had not been developed. In this study, we report the accumulation of fibroblasts and pronounced fibrotic remodeling, and an enrichment of fibrosis-associated gene signatures similar to bleomycin-induced pulmonary fibrosis in the lungs of hCD147 transgenic mouse model with SARS-CoV-2 infection. Compared with the hCD147 transgenic mice, the hACE2 mice cannot fully recapitulate the pathology of severe COVID-19, which features acute exudative alveolar pneumonia.^9^ In line with these findings, we observed that SARS-CoV-2 caused stronger fibrotic phenotypes in lungs of the hCD147 mouse model than in those of the hACE2 mouse model. Thus, our study highlights SARS-CoV-2-infected hCD147 mice as an ideal model for studying the fibrotic pathogenesis of COVID-19.

Given the severe clinical impact of COVID-19 compared to previous pandemic diseases such as SARS and MERS, the global health care system has already been challenged by patients with sequelae of long COVID.^38,39^ In a study to assess the sequelae of COVID-19 with a particular focus on the recovery of patients with non-severe COVID-19, survivors still had multi-system issues at the nearly 1-year follow-up.^40^ Chest CT evaluation showed that 56.7% patients showed abnormal CT findings, and fibrosis was one of the most common abnormal CT manifestations (17.5%).^40^ Therefore, it is important to develop urgently needed and effective therapies against pulmonary fibrosis. As antifibrotic agents that interfere with processes of pulmonary fibrosis, nintedanib and pirfenidone might provide novel therapeutic approaches for minimizing post-COVID-19 fibrosis.^41,42^ However, it is likely that nintedanib may increase the risk of bleeding when used together with anticoagulants.^43^ In addition, both nintedanib and pirfenidone may lead to hepatotoxicity, which will further increase the likelihood of liver dysfunction often experienced by COVID-19 patients.^44^ Inflammatory mediators have been proven to contribute to early organ injury in COVID-19, and anti-inflammatory treatments including anti-interleukin 6 (IL-6)/anti-IL-6R antibodies or janus kinase (JAK) inhibitors improve the clinical outcomes of severe COVID-19 when administered in the early phase.^45^ Considering early organ injury and strong immune responses are widely accepted as the initiation stage of pulmonary fibrosis, therapies targeting inflammatory mediators might be developed as early intervention strategies for patients at risk of developing fibrotic complications of COVID-19.

However, pneumonia, scarring and fibrosis were not only observed in patients with COVID-19 acute respiratory distress syndrome (ARDS), but also in individuals with initially mild or moderate disease.^46,47^ At the nearly 1-year follow-up, 16.7% of the non-severe cases suffered from pulmonary fibrosis, which was not significantly lower than that of severe cases, suggesting that the occurrence of pulmonary fibrosis is not totally associated with the severity of immune response to the viral infection.^40^ Thus, effective symptomatic treatments against the stages following inflammation when fibroblasts proliferate and the ECM is restructured need further exploration.

Our previous study has revealed that meplazumab, a humanized anti-CD147 antibody, effectively inhibited pulmonary inflammation caused by SARS-CoV-2 and its alpha and beta variants by eliminating viral entry and suppressing CyPA-mediated cytokine overproduction.^9^ In the phase II/III clinical trial, meplazumab treatment significantly reduced mortality of COVID-19 patients and increased live discharge without supplemental oxygen, suggesting the improvement of pulmonary function. Moreover, our study identified meplazumab as a promising drug against the accumulation of fibroblasts and production of ECM proteins, thus potentially alleviated the progression of pulmonary fibrosis caused by SARS-CoV-2 infection. Previous study has shown that CD147 promotes M1 macrophage polarization, which induces differentiation of Th17 cells and the progression of lung interstitial fibrosis.^48^ The impact of CD147 in SARS-CoV-2-induced pulmonary fibrosis may be not only due to its contribution to virus entry and immune responses, but also due to its role as a crucial regulator for fibroblasts activation. During liver fibrosis, CD147 was reported to promote the upregulation of fibrogenesis-related genes and stimulate HSC activation.^28,29^ Actually, a self-sustaining network between CD147 and the master mediator of myofibroblast differentiation and organ fibrosis, TGF-β, has been identified in both liver fibrosis and hepatocellular carcinoma progression.^29,49^ TGF-β stimulation could upregulate CD147 expression by PI3k-Snail-Slug signaling, and CD147 increases the transcriptional expression of TGF-β by β-catenin and activates latent TGF-β by matrix metalloproteinase (MMP).^49,50^ In this study, CD147 and TGF-β showed high expression levels on fibroblasts and macrophages in lung tissues of both SARS-CoV-2-infected hCD147 mice and classic pulmonary fibrosis model, giving rise to the possibility that the TGF-β1-CD147 positive feedback loop may also exist in the activation of fibroblasts during pulmonary fibrosis. As an extracellular MMPs inducer, CD147 is capable of promoting extracellular matrix remodeling by inducing the secretion of MMPs in fibroblasts and hepatic stellate cells.^28,51^ Elevated levels of MMPs, including MMP-1, MMP-2, MMP-3, MMP-7, MMP-8 and MMP-9 have been reported in idiopathic pulmonary fibrosis as well as in experimental fibrosis. Overexpression of MMP-2 and MMP-9 have been suspected to be implicated in basement membrane disruption, contributing to the destruction of structural integrity of the alveolar wall.^52^ A discontinuity of the basement membrane might also allow greater access for exudative factors and interstitial cells to the alveolar space, preceding further tissue destruction and the development of fibrosis.^53,54^ In addition, MMP-2 and MMP-9 have been shown to activate latent TGF- β1.^55,56^ In this study, we found that the humanized anti-CD147 antibody, meplazumab, markedly inhibited the elevation of both MMP-2 and MMP-9 in SARS-CoV-2-infected hCD147 mice. TGF-β stimulation upregulated the expression levels of MMP-2 and MMP-9 in cultured fibroblasts, which were suppressed by meplazumab. These data confirm the role of CD147 in promoting progressive fibrosis as a crucial MMPs inducer. These findings offer a possible explanation for the occurrence of pulmonary fibrosis in the non-severe COVID-19 cases.

In conclusion, our study provides an ideal model for studying the fibrotic pathogenesis of COVID-19, discovers a novel function of CD147 contributing to the activation of lung fibroblasts, and provides a promising drug against COVID-19 pulmonary fibrosis.

## Materials and methods

### Cell culture

MRC-5 cell line was purchased from Procell Life Science & Technology Co.,Ltd and has been authenticated using Short Tandem Repeat DNA profiling by Beijing Microread Genetics Co., Ltd (Beijing, China) and were cultured at 37 °C under 5% CO_2_ in DMEM medium supplemented with 10% fetal bovine serum (FBS), 1% penicillin/streptomycin, and 2% L-glutamine.

### CD147-RBD complex model construction and interaction profile analysis

The structure of CD147 (PDB ID: 3B5H) and RBD (PDB ID: 6M0J) was retrieved from Protein Data Bank respectively. CD147 − RBD docking were performed using the ZDOCK and RDOCK programs.^57,58^ Then the optimal docked complex was selected on the basis of energy and size of cluster and further refined by 1000 ns MD simulations using GROMACS 2018.2. The interaction residues were analyzed by PDBsum online server (http://www.ebi.ac.uk/thornton-srv/databases/pdbsum/Generate.html).

### Surface plasmon resonance (SPR)

The interaction between CD147 and the RBD of spike protein was analyzed by SPR assay performed by Biacore T200 SPR system (Cytiva) with CM5 sensor chip. Human His-CD147 and mouse His-CD147 were produced in our laboratory, and His-Spike RBD (40592-V08H) was from Sino Biological. RBD was fixed to the surface of sensor chips by amino coupling kit (GE Healthcare, BR-1000-50). The binding time was set to 90 s and dissociation time was 120 s. The results from the SPR system were analyzed by Biacore T200 Evaluation software 3.2 to determine the affinity constant.

### RNA-sequencing

Total RNA was extracted from lung tissues or MRC-5 cell line using Trizol reagent kit (Invitrogen) according to the manufacturer’s protocol. RNA quality was assessed on an Agilent 2100 Bioanalyzer (Agilent Technologies, Palo Alto, CA, USA) and checked using RNase free agarose gel electrophoresis. After total RNA was extracted, eukaryotic mRNA was enriched by Oligo (dT) beads. Then the enriched mRNA was fragmented into short fragments using fragmentation buffer and reverse transcripted into cDNA with random primers. Second-strand cDNA were synthesized by DNA polymerase I, RNase H, dNTP and buffer. Then the cDNA fragments were purified with QiaQuick PCR extraction kit (Qiagen, Venlo, The Netherlands), end repaired, poly(A) added, and ligated to Illumina sequencing adapters. The ligation products were size selected by agarose gel electrophoresis, PCR amplified, and sequenced using Illumina Novaseq6000 by Gene Denovo Biotechnology Co. (Guangzhou, China).

### Real-time quantitative PCR assay

For RT-PCR analysis, total RNA was isolated with a Total RNA Kit II (OMEGA, BioTek, D6934-01) in RNase-free conditions from cells lysed in RNA lysis buffer and then reverse transcribed into cDNA with RNA reverse transcriptase kit (TaKaRa, RR037A) according to the manufacturer’s protocol. RT-PCR was then performed with a TB Green PCR kit (TaKaRa, RR820A) using the ABI QuantStudio™ 7 Flex Real-Time PCR System (Applied Biosystems) to determine the expression levels of the genes of interest. The mouse organ tissues were homogenized to detect the virus gene copy number by qPCR (TaqMan™ Universal Master Mix II with UNG, 4440044, Applied Biosystems). All primers were synthesized by the Beijing Genomics Institute (Shenzhen, China). The primer sequences are listed in Supplementary Table 1.

### RNA interference

The MRC-5 cells were incubated with supernatant containing lentivirus carrying the shCD147 or shNT construct to generate CD147 knockdown cells. Non-Target shRNA Control Vector was obtained from Sigma (St. Louis, MO). The sequence for non-target shRNA control vector was: sense 5′-CGGCAACAAGATGAAGAGCACCAACTC-3′, anti-sense 5′-GAGTTGGTGCTCTTCATCTTGTTGTTTTT-3′. CD147 shRNA (corresponding nucleotide positions 352–373 of CD147 cDNA), sense 5′-CCCATCATACACTTCCTTCTT-3′, anti-sense 5′-AAGAAGGAAGTGTATGATGGG-3′. The small interfering RNA (siRNA) sequences targeting CD44s were designed and synthesized by Tsingke Biological Technology (Xi’an, China). The sequences were listed in Supplementary Table 2. The MRC-5 cells were transfected with the siRNAs using Lipofectamine 2000 (Invitrogen, Carlsbad, CA, US).

### Generation of fibroblast-specific CD147 knockout mice

C57BL/6-FSP1-CreERT2 transgenic mice were purchased from Shanghai Model Organisms Center. CD147 flox transgenic mice (CD147^f/f^) were cultured in our lab. The CD147^f/f^ mice were crossed with the FSP1-CreERT2 transgenic mice to obtain CD147^f/f^FSP1-Cre mice. Tamoxifen dissolved in coin oil was intraperitoneally injected at a dose of 75 mg/kg once every 24 h for a total of five consecutive days.

### SARS-CoV-2 infected hCD147 mice model and bleomycin-induced pulmonary fibrosis model

The animal experiments protocols used were approved by the Institutional Animal Care and Use Committee (IACUC) of Fourth Military Medical University (20200206, 20211102). Human CD147 (hCD147) mice were provided by Shanghai Model Organisms Center, Inc. (China). Human ACE2 (hACE2) mice were provided by the National Institutes for Food and Drug Control. After being anesthetized, each mouse was inoculated intranasally with SARS-CoV-2 at a dose of 3 × 10^5^ TCID_50_. At 2 d.p.i, lung tissues were collected for virus loads detection. For meplazumab treatment, 3 mg/kg meplazumab (produced by Jiangsu Pacific Meinuoke Biopharmceutical Co. Ltd) or human IgG was administered via the tail vein at 1 d.p.i. and 7 d.p.i. Tissues from the lung and other main organs were collected at indicated time points for RNA sequencing, PCR or fixed with paraformaldehyde and PLP Fixing Solution for H&E staining, IHC, immunofluorescence staining, and TEM. For bleomycin-induced pulmonary fibrosis model, male C57BL/6J mice, CD147^f/f^FSP1-Cre mice or CD147^f/f^ mice aged 10 weeks were selected as experimental subjects. After receiving anesthesia by an intraperitoneal injection of pentobarbital, mice were intratracheally injected with bleomycin dissolved in phosphate buffered saline (PBS) (total volume 20 μL, 4 mg/kg) or PBS. At different time points, the mice were euthanized by an intraperitoneal injection of pentobarbital and lung tissues were collected.

### Hematoxylin & Eosin (H&E) staining and Masson’s trichrome staining

Formalin-fixed paraffin-embedded mice main organ tissue sections were deparaffinized by xylene and alcohol. The slides were then counterstained with hematoxylin for 15 min and eosin for 10 min. Then the tissue sections were dehydrated and mounted in the resinous medium. Masson staining was performed according to the Masson’s trichrome staining Kit (Solarbio, G1340). The blue collagen tissue was visible under a light microscope. Images were analyzed with ImageJ Software.

### Detection of hydroxyproline in lungs

Lung tissue including the middle and lower lobes of the right lung was selected for examination. According to the manufacturer’s instructions (Abcam, ab222941), 10 N concentrated NaOH was added to samples and hydrolyzed at 120 °C for 1 h. After cooling on ice, samples were neutralized with 10 N concentrated HCl and centrifuged. The supernatant was collected and samples and standards were added to wells. Wells were evaporated to dryness by heating at 65 °C. Then oxidation reagent was added to dissolve crystalline residue, and incubated at room temp for 20 min. The Developer was added incubated at 37 °C for 5 min, and DMAB concentrate was added and incubated for 45 min at 65 °C. After the reaction, the supernatant could be used to detect the absorbance value at 560 nm wavelength.

### Transmission electron microscopy

The organ tissues were cut into 1 mm^3^ pieces and fixed with 4% glutaraldehyde fixing solution for at least 24 h. After washing with PBS, the sections were treated with osmic acid for 1.5 h. Tissues were then dehydrated with alcohol at gradient concentrations and soaked in acetone for 15 min. After embedding and polymerization overnight with epoxy resin, the slices were cut with an ultrathin slicer and glued to a perforated copper grid. Section was stained with lead citrate and uranium solution for 7 min, washed with water, and dried. The ultrastructure was observed by transmission electron microscopy (HT7800, Hitachi).

### Multiplex immunohistochemistry staining (mIHC)

In brief, slides were dewaxed, followed by antigen retrieval in citrate (pH = 6) or Tris-EDTA (pH = 9) buffer. After blocked with 5% goat serum, the slide was sequentially incubated with primary antibodies recognizing α-SMA (Abcam, ab7817), CD3 (Abcam, ab5690), CD4 (Abcam, ab183685), CD8 (Boster Bio, a02236-1), CD19 (Abcam, ab245235), Ly6G (Abcam, ab238132), NCR1 (Abcam, ab233558), F4/80 (Cell Signaling Technology, 70076 S), TGF-β (Abcam, ab215715), Sftpc (Abcam, ab90716), rabbit anti-mouse CD147 (Abcam, ab188190), mouse anti-human CD147 (prepared in our own lab), FSP1 (Proteintech, 20886-1-AP), rabbit IgG Isotype Control (Invitrogen, 31235), mouse IgG Isotype Control (Invitrogen, 14-4714-82). Goat anti-Mouse F(ab) fragment was used for mouse-on-mouse blocking. TSA-Indirect Kit (PerkinElmer, USA) was used according to the manufacturer’s manual. Images were analyzed with HALO^™^ Image Analysis Software or ImageJ Software.

### Flow cytometric analysis

Disaggregated single cells were isolated from lungs for flow cytometric analysis. Antibodies used were PE anti-CD147 (Biolegend, 123707), α-SMA (Abcam, ab7817), goat anti-mouse IgG (H + L) secondary antibody Dylight 594 (Invitrogen, 35510). The data was acquired with a BD Fortessa flow cytometer with BD FACSDiVa™ software and analyzed by FlowJo, v.10.5.3.

### Statistical analysis

Data were presented as the mean ± SD unless otherwise indicated. A two-tailed Student’s *t*-test was used to compare means between two groups. For analysis of differences between the groups, one-way ANOVA for individual comparisons between the groups was performed. Statistical analysis was performed using GraphPad Prism 7.0 (GraphPad Prism Software, CA, USA). Differences were considered to be significant when *P* < 0.05.

## Supplementary information

Supplementary Figures and Tables

## Data Availability

The RNA-seq data generated in this study have been deposited in the NCBI Sequence Read Archive (SRA) database under the accession code PRJNA816656, PRJNA817727 and PRJNA817626. Other data sets in this study are available from the corresponding author upon reasonable request.
