# Supplementary material for: CD147 contributes to SARS-CoV-2-induced pulmonary fibrosis
Source: Signal Transduct Target Ther. 2022 Nov 25;7:382. doi: 10.1038/s41392-022-01230-5 (PMC9691700; doi:10.1038/s41392-022-01230-5)
Supplement: Supplementary file 1 — Supplementary Figures and Tables [file 41392_2022_1230_MOESM1_ESM.pdf]

Supplementary Materials for
CD147 contributes to SARS-CoV-2-induced pulmonary fibrosis

Jiao Wu[1], Liang Chen[2], Chuan Qin[3], Fei Huo[1], Xue Liang[1], Xu Yang[1], Kui Zhang[1], Peng Lin[1], Youning Liu[3], Zhuan Feng[1], Jiansheng Zhou[1], Zhuo Pei[1], Yatao Wang[1], Xiuxuan Sun[1], Ke Wang[1], Jiejie Geng[1], Zhaohui Zheng[1], Xianghui Fu[1], Man Liu[1], Qingyi Wang[1], Zheng Zhang[1*], Huijie Bian[1*], Ping Zhu[1*], Zhi-Nan Chen[1*]

*Correspondence: Zheng Zhang (zhangzzy@aliyun.com) or Huijie Bian (hjbian@fmmu.edu.cn) or Ping Zhu (zhuping@fmmu.edu.cn) or Zhi-Nan Chen (znchen@fmmu.edu.cn)

This PDF file includes:

Supplementary Figures 1 to 8

Supplementary Table 1 to 2

Supplementary Figure 1

a

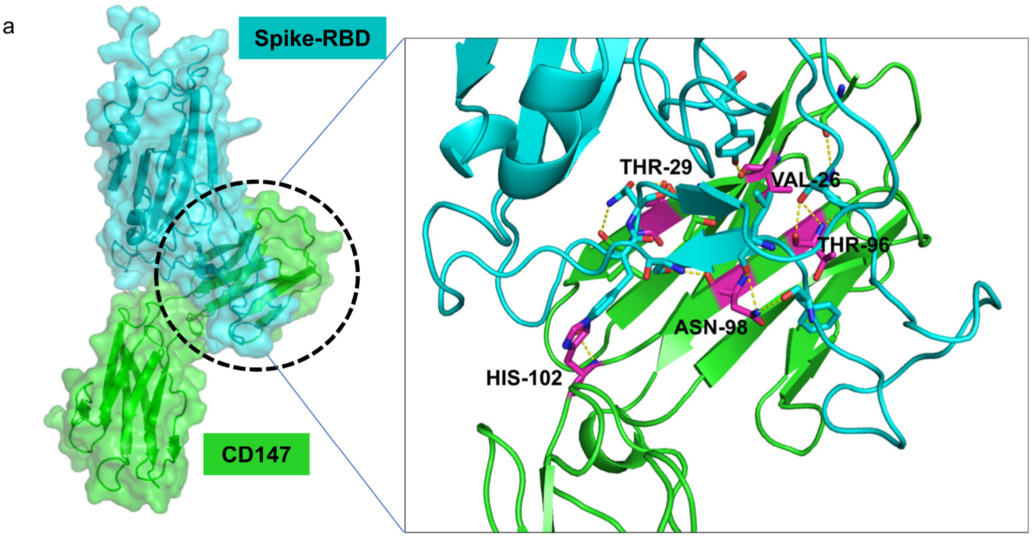

Binding free energies (ΔG_{bind}): -27.50 kcal/mol

b

	CD147			Spike-RBD		
	Residue	No.	< -- >	Residue	No.	Distance
1	GLY	24	< -- >	THR	464	3.14
2	VAL	26	< -- >	TYR	345	2.56
3	THR	28	< -- >	ASN	444	3.12
4	THR	29	< -- >	ASN	444	2.98
5	VAL	30	< -- >	ASN	444	2.94
6	THR	96	< -- >	THR	464	2.66
7	THR	96	< -- >	THR	464	2.91
8	THR	96	< -- >	THR	464	2.56
9	ASN	98	< -- >	LEU	486	2.78
10	ASN	98	< -- >	SER	488	3.07
11	ASN	98	< -- >	PHE	484	2.79
12	ASN	98	< -- >	LEU	486	2.86
13	HIS	102	< -- >	TYR	443	3.05

c

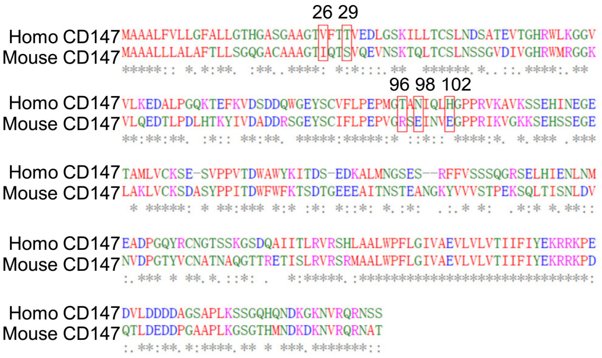

d

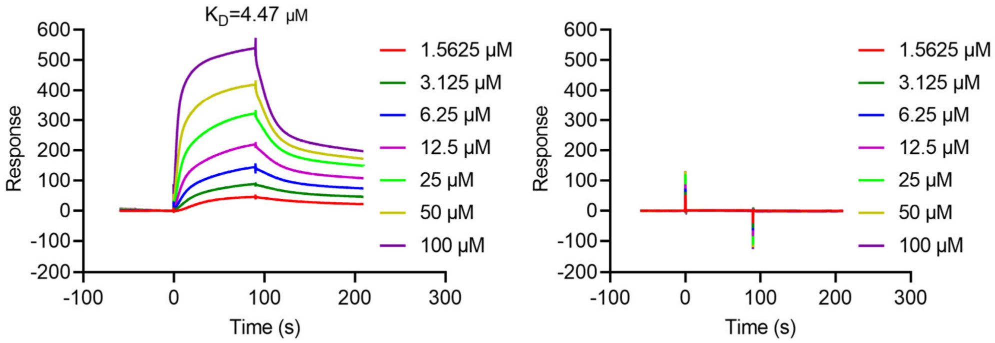

Supplementary Fig. 1 Binding mode between the RBD of the SARS-CoV-2 spike protein and CD147. **a** Left, Surface representation of the proposed complex. RBD of the spike protein and CD147 were shown in cyan and green respectively. Right, Polar interactions at the interface. Key interacting residues were shown as sticks and the residues differ between human and mouse CD147 were marked in pink. **b** Specific residues that formed hydrogen bonds. Gray background color indicated the different

residues between human and mouse. **c** Sequence alignment of human and mouse CD147. Red boxes indicated the residues involved in hydrogen bonds formation. **d** The interaction of human (left) or mouse (right) CD147 and RBD of the spike protein was detected by SPR assay.

Supplementary Figure 2

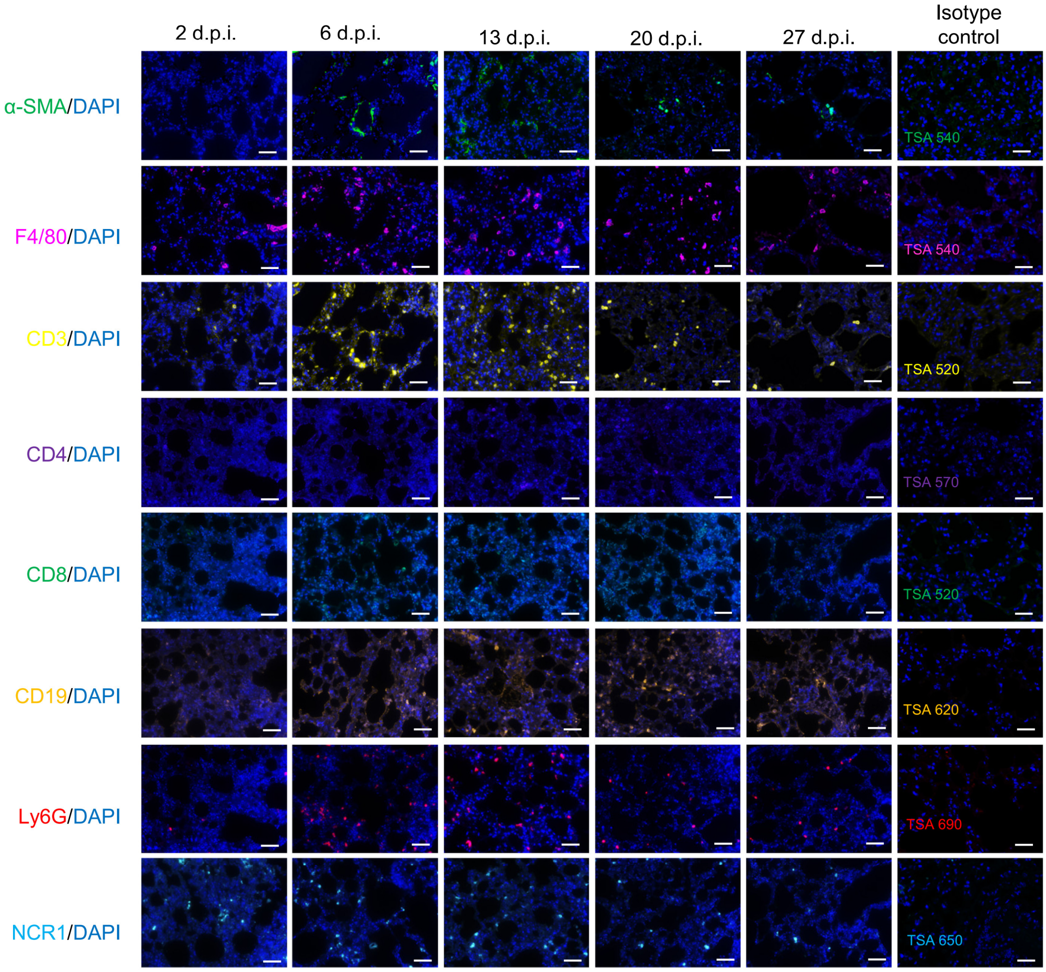

Supplementary Fig. 2 hCD147 transgenic mice infected with SARS-CoV-2 showed accumulation of inflammatory cells and expansion of fibroblasts in lung tissues. Representative multiplex immunohistochemistry (mIHC) staining of lung tissue sections for α-SMA, F4/80, CD3, CD4, CD8, CD19, Ly6G, and NCR1 from SARS-CoV-2-infected hCD147 mice at indicated time points. Several multiplex panels were assembled to show all these targets. The isotype control (10 μg/mL of rabbit IgG isotype control or mouse IgG isotype control) was used along with the staining of different molecules. Scale bars, 100 μm.

Supplementary Figure 3

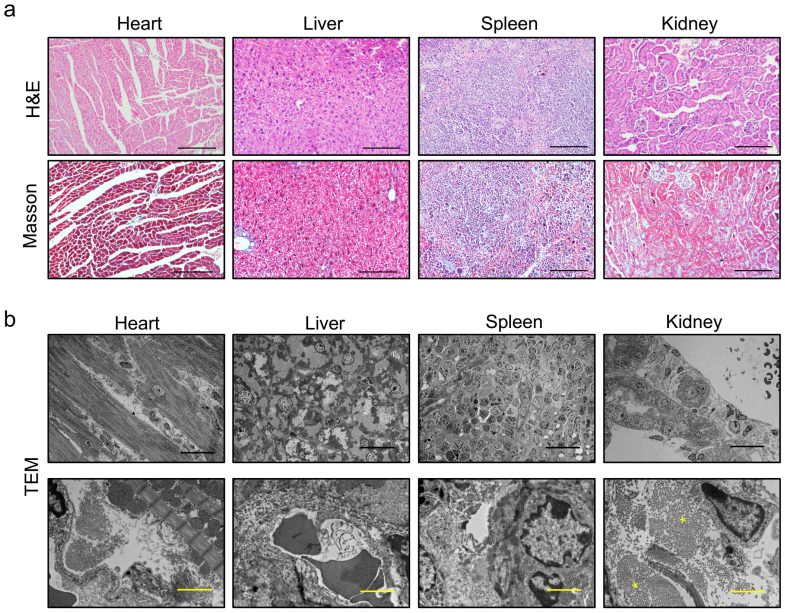

Supplementary Fig. 3 Pathological changes in organs of hCD147 transgenic mice infected with SARS-CoV-2. **a** H&E staining images of representative hearts, livers, spleens, lungs and kidneys in SARS-CoV-2-transfected hCD147 transgenic mice at 13 d.p.i. Scale bars, 200 μm. **b** TEM analysis of main organs. Black scale bars, 20 μm; yellow scale bars, 2 μm. The stars indicate collagen fibrils.

Supplementary Figure 4

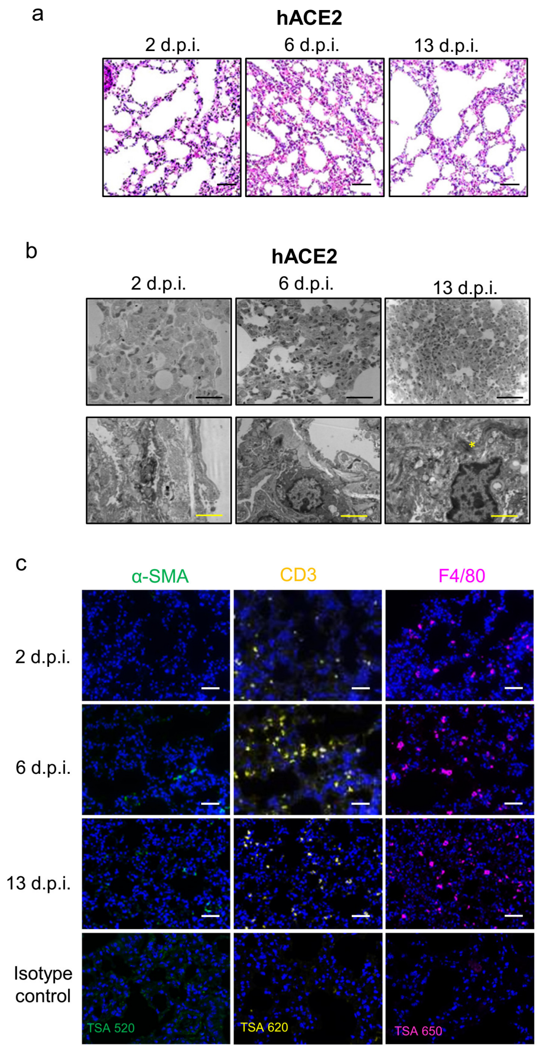

Supplementary Fig. 4 Pathology and fibrotic features of hACE2 transgenic mice infected with SARS-CoV-2. **a** H&E staining images of hACE2 transgenic mice infected with SARS-CoV-2. Scale bars, 100 μm. **b** TEM analysis of lung tissues at indicated time points. Black scale bars, 20 μm; yellow scale bars, 2 μm. The stars indicate collagen fibrils. **c** Multiplex immunohistochemistry of lung tissue sections for α-SMA, F4/80 and CD3. The rabbit IgG isotype control or mouse IgG isotype control was used along with the staining of different molecules. Scale bars, 100 μm.

Supplementary Figure 5

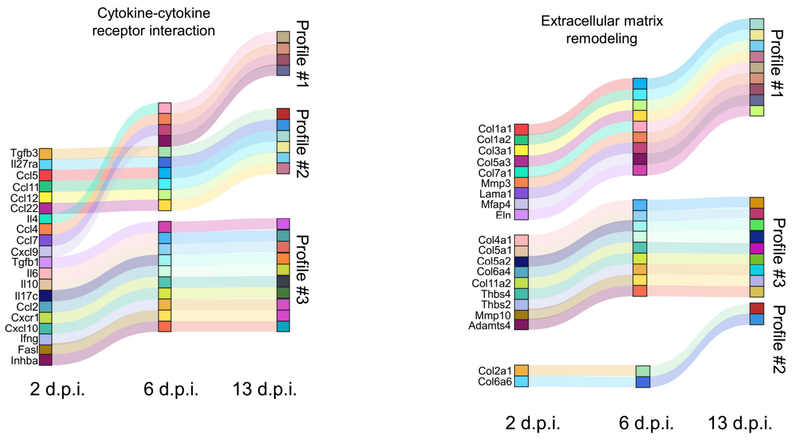

Supplementary Fig. 5 The statistically significant profiles by trend analysis of differentially expressed genes in lungs of SARS-CoV-2-infected hCD147 mice. Sankey diagrams of significantly enriched genes associated with fibrosis involved in cytokine-cytokine receptor interaction and ECM remodeling in the three profiles. Profile #1 (continuously increasing from 2 to 13 d.p.i.), profile #2 (starting the increase from 6 d.p.i.), profile #3 (reaching the plateau at 6 d.p.i.).

Supplementary Figure 6

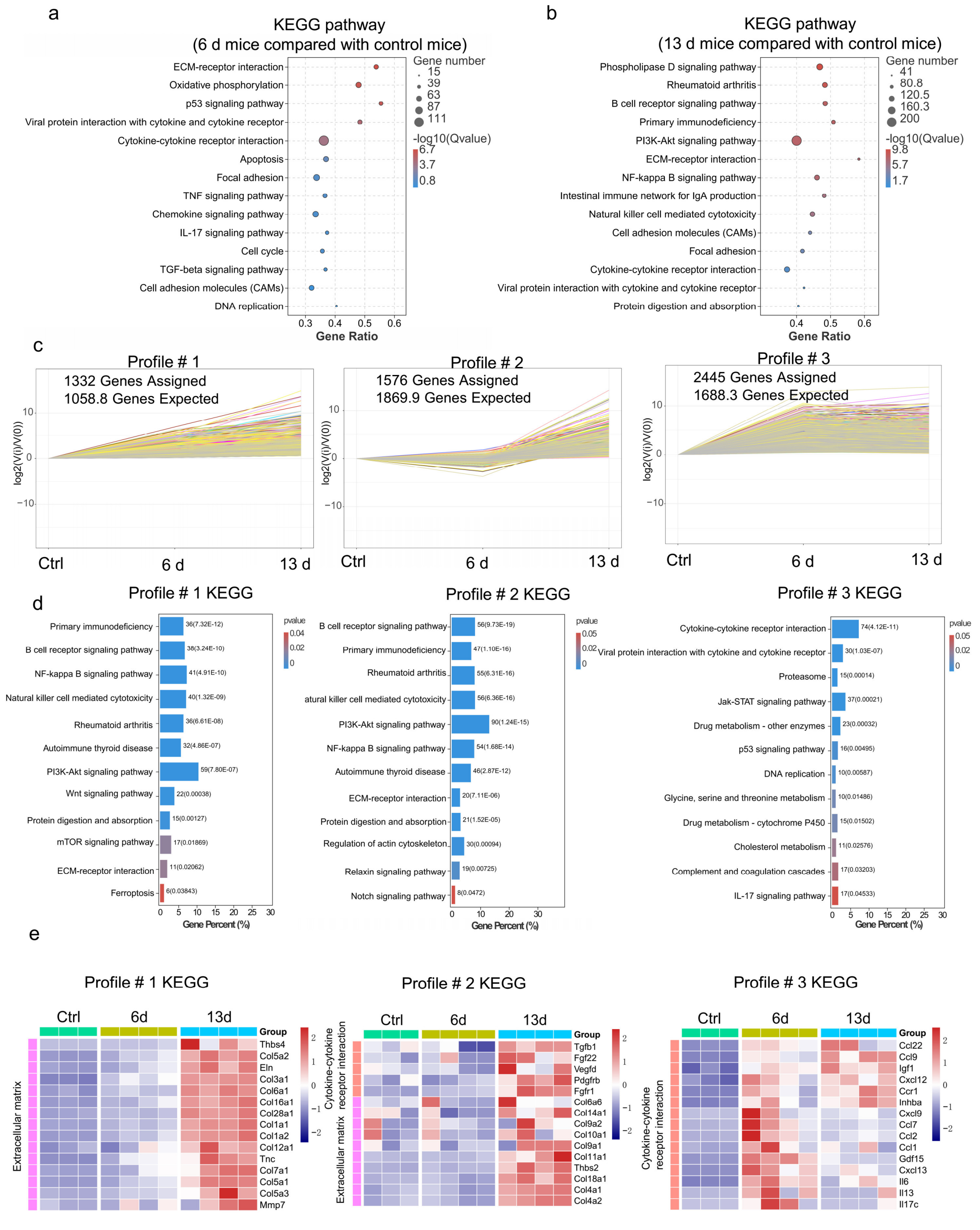

Supplementary Fig. 6 Transcriptional signatures of bleomycin-induced pulmonary fibrosis revealed by RNA-seq analysis.

KEGG enrichment analysis of pathways enriched in differentially expressed genes in lungs of bleomycin-induced mice at 6 days (**a**) and 13 days (**b**) compared with control mice**. c** Statistically significant profiles by trend analysis of differentially expressed genes in normal lung tissues and at 6 and 13 days. Profile #1, continuously increasing from control to 13 days. Profile #2, starting the increase from 6 days. Profile #3,

reaching the plateau at 6 days. **d** KEGG enrichment analysis of pathways enriched in the three profiles. **e** Heatmap of significantly upregulated genes involved in cytokine-cytokine receptor interaction and ECM remodeling in the three profiles.

Supplementary 7

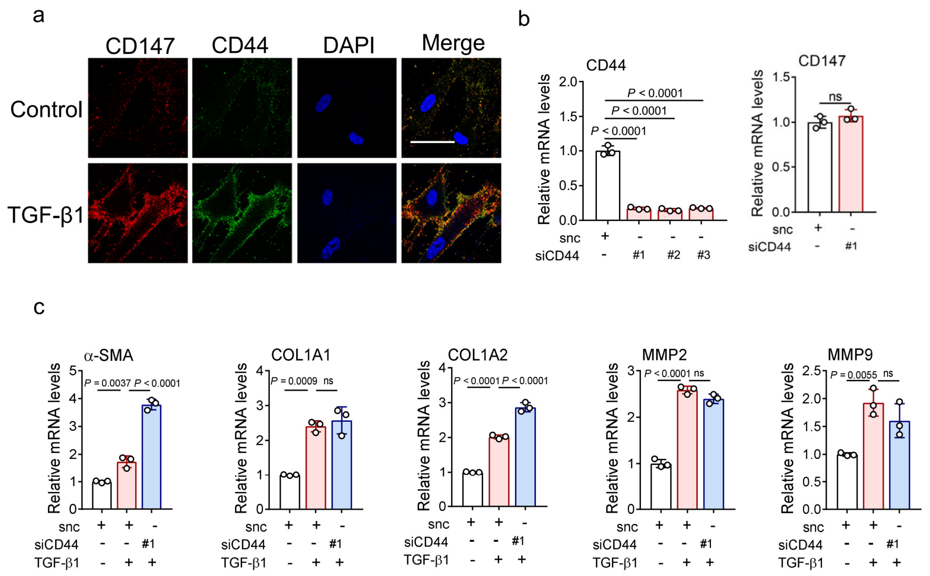

Supplementary Fig. 7 The role of CD44 in TGF-β-CD147 axis in fibroblast activation. **a** MRC-5 cells were stimulated with TGF-β (10 ng/mL) and the co-localization of CD147 and CD44 (primary antibody: Cell Signaling Technology, 3570) was detected by immunofluorescence. Scale bars, 20 μm. **b** The mRNA levels of CD147 and CD44 in MRC-5 cells transiently transfected with siRNA for CD44 gene silence. n = 3 samples for each group. Left, one-way ANOVA followed by multiple comparisons. Right, two-tailed unpaired t test. **c** MRC-5 cells were transfected with siRNA for CD44 gene silence and stimulated with TGF-β. The gene expression levels of α-SMA, COL1A1, COL1A2, MMP2 and MMP9 were detected by real-time PCR. n = 3 samples for each group, one-way ANOVA followed by multiple comparisons.

Supplementary Figure 8

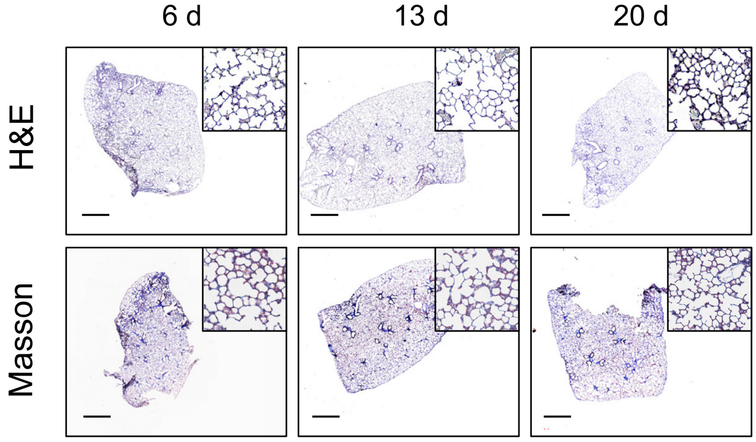

Supplementary Fig. 8 Pathological changes in lungs of CD147$^{f/f}$ mice treated with PBS. H&E staining and Masson's trichrome staining images of representative lungs in CD147$^{f/f}$ mice receiving 50 μl of PBS. Scale bars, 1 mm.

Supplementary Table 1 The primers for qPCR

**Gene name**	**Forward primer (5' - 3)**	**Reverse primer (5' - 3')**
SARS-CoV-2 N protein	GGGGAACTTCTCCTGCTAGAAT	CAGACATTTTGCTCTCAAGCTG
SARS-CoV-2 N protein probe	FAM-TTGCTGCTGCTTGACAGATT-BHQ1	
Mouse TGF-β	CGGCAGCTGTACATTGACTT	CCGGGTTGTGTTGGTTGTAG
Mouse PDGF	GGTCCAGGTGAGGTTAGAGG	GAGGAGAACAAAGACCGCAC
Mouse MMP2	ATAGAGACTGGCTTAGGAGG	GGCATACAAAAGCAACAGTT
Mouse MMP9	TTAAGGTATTCAGTTGCCCC	TTTACAGGACACGGAGAATC
Mouse TIMP1	CCTAAGGAACGGAAATTTGC	CCAGTTTGCAAGGGATAGAT
Mouse TIMP2	ATTAAGACATATCCGTGGGC	TGCTGACTTTCAACAACTCT
Mouse COL1A1	CCTCAGGGTATTGCTGGACA	TCCCTCACGTCCAGATTCAC
Mouse COL1A2	TGATGTGCATTGTGTGTGAT	TGGGCAAGAAGGAAAATGAG
Mouse COL3A1	CTAAACACACTGGGGAATGG	GGTTTCAGAGAGTTTGGCTT
Mouse GAPDH	TAAATACGGACTGCAGCCCT	CATTCTCGGCCTTGACTGTG
Human α-SMA	CTATGAGGGCTATGCCTTGCC	GCTCAGCAGTAGTAACGAAGGA
Human CD147	GGAACTCTTCCTGAGGTGGC	CTCCCGGGCTCTCGTGAAAC
Human CD44	CCAGAAGGAACAGTGGTTTGGC	ACTGTCCTCTGGGCTTGGTGTT
Human COL1A1	GGGAATCACTGGTGCTATAG	GCTCTCCTCCCATGTTAAAT
Human COL1A2	GAGTTGTATCGTGTGGTGTA	TGGAACCATGGAAGAAGATG
Human MMP2	AGCGAGTGGATGCCGCCTTTAA	CATTCCAGGCATCTGCGATGAG
Human MMP9	GCTACGTGACCTATGACATC	AACAAAGGTGAGAAGAGAGG
Human GAPDH	GCACCGTCAAGGCTGAGAAC	TGGTGAAGACGCCAGTGGA

Supplementary Table 2 The sequences for RNA interference.

siRNA	Sense	Antisense
si-CD44#1	CUCCCAGUAUGACACAUAUTT	AUAUGUGUCAUACUGGGAGTT
si-CD44#2	GGACCAAUUACCAUAACUATT	UAGUUAUGGUAAUUGGUCCTT
si-CD44#3	GCAGUCAACAGUCGAAGAATT	UUCUUCGACUGUUGACUGCTT
